# Clinical and histopathological pattern of lung cancer in Morocco

**DOI:** 10.11604/pamj.2022.42.283.35593

**Published:** 2022-08-16

**Authors:** Ouassima Erefai, Abdelmajid Soulaymani, Abdelrhani Mokhtari, Hinde Hami

**Affiliations:** 1 Laboratory of Biology and Health, Faculty of Science, Ibn Tofail University, Kenitra, Morocco

**Keywords:** Lung neoplasms, epidemiology, adenocarcinoma, Morocco

## Abstract

**Introduction:**

lung cancer is the most common cancer and the leading cause of cancer death worldwide. This study aimed to provide an overview of the epidemiology of primary lung cancer in Morocco. The distribution of histological subtypes by sex and smoking status was also assessed.

**Methods:**

this was a retrospective and descriptive study using medical records of patients with primary lung cancer, diagnosed at two university hospitals in Morocco between 2014 and 2017.

**Results:**

a total of 606 patients (average age = 58.5 ± 10.64 years, men = 521) were included. Four hundred and forty-three men had a history of smoking against sex women. Most patients (85.68%) had respiratory symptoms at diagnosis. Over half of patients (53.03%) had a performance status <2 and 38.94% had at least another pulmonary disease at presentation. Chronic obstructive pulmonary disease (COPD) and tuberculosis were present in 23.43% and 18% of patients, respectively. The majority (72.27%) of men practiced an occupation associated with a significant risk of lung cancer. Adenocarcinoma was the main histological type in our series with 60.40%. Most (79.55%) patients were diagnosed at stage IV. Only 7.83% of patients benefited from surgery. The distribution of histological subtypes by sex and smoking habits showed that adenocarcinoma was more frequent in women (p=0.011), and squamous cell carcinoma in men (p=0.014). No differences between smokers and non-smokers were noted.

**Conclusion:**

our results showed a decrease in the age of diagnosis and a late stage of the disease. Adenocarcinoma was the most frequent histological type.

## Introduction

Lung cancer is the most commonly diagnosed cancer and the leading cause of cancer death in the world [1], with about 2.2 million new cases and 1.8 deaths reported in 2020 [2]. In Africa, the incidence of lung cancer is on the rise, especially in North Africa [1,3,4]. Morocco has an important incidence rate and the highest mortality rates in Africa and Middle East and Northern Africa (MENA) region, with 7,353 new cases and 6,551 deaths in 2020 [5,6]. Since the first studies published in 1950, a strong association between smoking and lung cancer was observed [7]. The incidence of lung cancer in a population is highly related to prevalent smoking habits [8]. Tobacco smoking has been reported to be responsible for more than 85% of lung cancer deaths [5]. In addition to tobacco, other etiologies have been related to lung cancer [9-12]. Studies have demonstrated that lung diseases, especially chronic obstructive pulmonary disease (COPD) and tuberculosis are related to a higher risk of lung cancer even in non-smokers [9]. A significant proportion of lung cancer is attributable to occupational carcinogens [10]. Other factors, such as passive smoking, air pollution, family history of lung cancer, and an unbalanced diet are increasingly recognized [11,12]. During the last 50 years, changes have been observed in the histologic characteristics of lung cancer, with an increasing proportion of adenocarcinoma [13]. This rise is likely explained by the changes in the design and composition of cigarettes, as well as the smokers´ behaviors [14]. Also, increased trends in incidence in women have been noted especially in developed countries, known for their high female tobacco consumption [15]. The patterns and trends in lung cancer epidemiology have changed considerably over the last decades worldwide [16]. In Morocco, studies on lung cancer are very limited. Identification of demographic, clinical, and histopathological characteristics, as well as exposure to known risk factors for patients with primary lung cancer, is an important step toward a better understanding and control of this disease. The main objective of this study was to provide an overview of the epidemiology of primary lung cancer in Morocco. The distribution of histological subtypes by sex and smoking status was also assessed.

## Methods

**Study design and population:** this retrospective study was performed at two university hospitals in Rabat (capital of Morocco), Ibn Sina, and Moulay Youssef Hospitals. Patients diagnosed with primary lung cancer from January 1, 2014, to December 31, 2017, were included. All diagnosis of primary lung cancer was proved by anatomopathological analysis of biopsy specimen. Patients with other lung diseases, metastatic lung cancer from other organs, or who did not provide complete information were excluded from this study.

**Data collection and analysis:** the hospital charts were reviewed. The following data were collected: age at diagnosis, sex, occupation, health insurance, smoking habits, clinical respiratory symptoms, eastern cooperative oncology group (ECOG) performance status, previous respiratory disease, personal history of cancer, familial history of cancer, pathology method, clinical stage, histological type, metastatic sites, and therapeutic protocol. All data were analyzed using Jamovi version 2.0.0. Quantitative variables were expressed in mean and standard deviation. Qualitative variables in frequencies and percentage. Khi-2 test was used to compare qualitative variables. A p-value < 0.05 was statically significant.

## Results

In total, 606 patients diagnosed with primary lung cancer were included in our study. The demographic and clinical characteristics of the study population are listed in Table 1. The average age at diagnosis was 58.5 years ±10.64 (19-85 years). The majority (73.2%) were <65 years. The 55-64 age group was the most represented (42.1%). A clear male predominance was observed with 85.9%. The presence of respiratory signs was the main reason for consultation. Thus, 81.17% of the patients consulted because of one or more respiratory symptoms, 14.63% for an extrathoracic problem, and 4.20% were asymptomatic. In asymptomatic cases, the discovery of lung cancer was incidental, as a result of a pathological X-ray during an investigation for another disease. Of patients with extrathoracic problems, 81.25% had altered general condition, and 11.25% had bone pain. Regarding the distribution according to performance status, over half (53.03%) of patients had a performance status score of less than 2. The smoking habits and exposure to other known risk factors by gender are summarized in Table 2. In men, never-smokers represented 14.97%, with current and former smokers comprising 74.28% and 10.75% respectively. Of these, the majority were moderate to heavy smokers with consumption of ≥20 pack-years in 82.62% of cases. More than two-thirds (67.7%) started smoking at a younger age (≥20 years). Only sex women were smokers, with a mean consumption of 36.15±15.51 pack-years. Regarding the exposure to the other known risk factors, over a third (38.94%) of cases had at least one pre-existing non-malignant pulmonary disease. Chronic obstructive pulmonary disease was reported in 23.43% of cases. Of these, 17.86% had never smoked. Pulmonary tuberculosis was observed in 18% of patients, of which 34.37% were never-smokers. Pneumonia was present in 6.60% of patients. The study of occupational and domestic exposure to risk factors showed that 71.57% of men were exposed to carcinogens in their work environment. Seven non-smoking women reported their exposure to smoke from coal. Another cancer diagnosed before the diagnosis of lung cancer was mentioned in thirteen patients. All of these cases had received radiation therapy while being treated for these cancers. Seven patients had a family history of cancer. The tumor characteristics are shown in Table 3. In the totality of cases, 84.30% were non-small cell lung cancer (NSCLC). The most important subtype in our series was adenocarcinoma, followed by squamous cell carcinoma with respectively 60.40% and 22.8% of cases. The distribution of histological subtypes by sex and smoking habits showed that adenocarcinoma was more frequent in women (77.92% versus 63.1%) (p=0.011), whereas squamous cell carcinoma was more prevalent in men (26.39% versus 12.98%) (p=0.010). No differences in histological types were noted between smokers and non-smokers. More than two-thirds of patients (79.55%) were diagnosed at stage IV. Almost all (92.10%) of the study population had at least one metastasis to one or more organs. The most affected organs were the thorax (48.53) and bone (18.4%). Among the 606 patients in our series, 39 cases did not receive treatment because of death or refusal of treatment. Concerning therapeutic management, only 31 (7.83%) patients benefited from surgery.

**Table 1:** distribution of demographic and clinical characteristics of the study population

Characteristics	No cases	%
**Gender (N=606)**		
Men	521	85.97
Women	85	14.03
**Age group (N=606)**		
<45	50	8.25
45-54	138	22.8
55-64	255	42.1
65-74	117	19.3
>75	46	7.55
**Health insurance (N=606)**		
Yes	478	78.88
No	128	21.12
**Respiratory symptoms (N=606)**		
Cough	351	57.92
Dyspnea	249	41.10
Hemoptysis	150	24.80
**Performance status (N=445)**		
0-1	236	53.03
2-4	209	46.97
**Therapeutic protocol (N=396)**		
Radiotherapy/chemotherapy	218	55.05
Palliative care	53	13.83
Surgery	31	7.83

**Table 2:** exposure to the known risk factors in the study population, by gender

	Men	Women	Total
**Smoking habits (N=606)**			
Smoking status n (%)			
Never smokers	78 (14.97)	79 (92.94)	157 (25.91)
Smokers	443 (85.03)	6 (7.06)	449 (74.09)
Cumulative consumption (pack years)	36.20 ±15.52	35.24 ± 12.72	36.15 ± 15.51
Light smokers <20	57 (17.38)	0 (0)	57 (17.17)
Moderate smokers 20-40	173 (52.74)	6 (100)	179 (53.31)
Heavy smokers >40	98 (29.88)	0 (0)	98 (29.52)
**Exposition to professional carcinogens (N=478)**			
Masonry/construction	107 (27.1)	0 (0)	107 (22.38)
Agriculture	70 (17.72)	1 (1.2)	71 (14.85)
Transportation	103 (26.07)	0 (0)	103 (21.55)
**Previous pulmonary disease (N=606)**			
COPD	127 (20.95)	15 (17.64)	142 (23.43)
Tuberculosis	92 (17.66)	17 (20)	109 (18)
Pneumonia	24 (4.6)	16 (18.82)	40 (6.6)
**History of cancer (N=102)**			
Personal history	7 (8.23)	6 (35.3)	13 (12.74)
Family history	4 (4.7)	3 (17.65)	7 (6.86)

COPD: chronic obstructive pulmonary disease

**Table 3:** tumor characteristics of the study population

Characteristics	No cases	%
**Pathology method (N=606)**		
CT-guided biopsy	285	47.03
Bronchoscopy	194	32.01
Pleural biopsy	52	8.58
Others	75	12.38
**Histological type (N=606)**		
Adenocarcinoma	366	60.40
Squamous cell carcinoma	138	22.80
Neuroendocrine tumor	55	9.1
Others types	47	7.7
**Stages (N=359)**		
I-II	27	7.52
III-IV	332	92.48
**Metastasis sites (N=443)**		
Thoracic lymph node	197	44.47
Bone	76	17.15
Pleura	62	14
Liver	42	9.48

CT-guided biopsy: computed tomography guided biopsy

## Discussion

During four years of this study, a total of 606 cases of primary lung cancer were diagnosed at the study sites. This reflects the high occurrence of lung cancer in our country. Morocco is one of the most affected countries in Africa by this disease [5]. While lung cancer mainly affects the elderly, and its risk increases with age [1,8,10], only 26.85% of patients in our series were over 64 years. The mean age at diagnosis was 58.5 years, whereas the corresponding figures of Rabat were 61.24 years in 2009-2011 [17], showing a decrease in the age at diagnosis among patients with lung cancer. Moreover, we found the lowest average age reported in the literature for Moroccan regions and North Africa, with 59 years in Marrakech, 59.1 years in Eastern Morocco, 62.06 in Algeria, and 61 years in Sousse (Tunisia) [18-21]. In France, the average age was 65.5 years [22]. Based on this observation, we could hypothesize that exposure to lung cancer risk factors is occurring in early life in the population of Rabat. Historically, lung cancer affected more men than women, but with changes in smoking and work habits, lung cancer is increasing among women, especially in developed countries [10]. The male-to-female ratio varies considerably from one region to another, ranging from 1.2 in Northern America to 5.6 in Northern Africa [2]. A French study noted an increase in the proportion of women with primary lung cancer from 16% to 24.3% in 10 years [22]. Regarding lung cancer mortality, more men than women die each year due to this disease, but the gap between the two sexes is narrowing, and it is expected that the number of female deaths will exceed those of males by 2045 [23]. Our study showed a net male predominance (86%), lower than that noted in Tunisia (92%) [19], but close to that found in Algeria (84%) [20]. Of the 606 patients included in our study, 14.03% of cases were female over 12.6% and 9.9% were found respectively in 2005 and 2006-2008 in Rabat [24,25].

Thus, lung cancer is increasing among Moroccan women, which will further increase the burden caused by this pathology. As we have already indicated, adenocarcinoma is on the rise compared to squamous cell carcinoma, especially in developed countries and in women [7,15]. Our series follows this trend. Adenocarcinoma was the most important subtype of lung cancer and was more frequent in women. In other regions of Morocco, such as Casablanca and Marrakech, squamous cell carcinoma was the most prevalent subtype [26,27]. Almost all the men were smokers. The majority were heavy smokers and started to smoke at a young age. A strong dose-response association between cumulative cigarette consumption and a higher risk of death from this disease has been reported [10]. Also, an inverse correlation between the age of smoking onset and increased lung deoxyribonucleic acid (DNA) damage has been shown [28]. People who started smoking before the age of 20 years have a higher risk to develop lung cancer [29]. Non malignant lung diseases, such as COPD and tuberculosis, have been reported to be associated with a high risk of lung cancer [11]. These diseases were largely present in our series. A study found that COPD was responsible for, respectively, 10% and 12% of lung cancer cases among never and heavy smokers [30]. Another study has shown that tuberculosis contributed to a significant incidence of lung cancer even in non-smokers with an attributable fraction in China, where tuberculosis is prevalent, of 12.67%, and in North America, where tuberculosis prevalence was lowest, at 1.14% [14]. Morocco is among the countries most affected by these diseases. Lung cancer is one of the cancers most associated with occupational exposure [12]. The majority of the cases studied were practicing occupational activities related to a significant risk of lung cancer. Occupational exposures among construction workers and farmers are highly associated with lung cancer risk [31,32]. Also, exposure to vehicle exhaust seems to play a crucial role in lung cancer development [33]. Regarding exposure to coal smoke, a meta-analysis found a consistent association between coal use and an increased risk of lung cancer [34]. United state has seen, that there are many known risk factors for lung cancer. Tobacco use is by far the main risk factor for this disease [22]. In Morocco, the tobacco control legislation of 1996 was insufficient to reduce the prevalence of smokers, which increased from 17.2% to 18.5% among adults between 2000 and 2006 [35]. In addition, smoking was responsible for 87% of lung cancer cases [36]. For this, effective tobacco programs are important to reduce the burden of lung cancer. Countries with strong antitobacco policies, such as Australia, California, and Hong Kong are more likely to have continued declines in lung cancer incidence than other countries, such as China, without coordinated tobacco control programs [4].

**Limitations:** the main limitation of this study is its retrospective nature. The absence of some specific information from the patient files did not allow the study of aspects such as exposure to passive smoking among women.

## Conclusion

Lung cancer is a serious public health problem in Morocco. According to our results, the epidemiological profile of lung cancer in Morocco is characterized by a high frequency of adenocarcinoma and cases diagnosed at advanced stages. In addition, younger age at diagnosis and an increased incidence of lung cancer in women were observed. Tobacco remains by far the most present risk factor in men with primary lung cancer, underscoring the need for a comprehensive lung cancer control policy including strong antitobacco programs, efforts to decrease exposure to other risk factors, early detection, and targeted strategies for the population at highest risk of lung cancer.

### What is known about this topic

- Lung cancer is the most frequent cancer in Morocco;
- Little is known about the epidemiology of lung cancer in Morocco.

### What this study adds

- This study provides a general overview of the epidemiology of lung cancer in Morocco;
- The results demonstrate a decrease in the age at diagnosis, an increase of female cases, and a late stage of the disease. Adenocarcinoma is the most important histological subtype.
